# Hypertrophic cardiomyopathy-related left ventricular pseudoaneurysm: A case report

**DOI:** 10.1016/j.heliyon.2024.e32197

**Published:** 2024-05-31

**Authors:** Katsuya Hashimoto, Hiroyuki Yamamoto, Atsushi Harada, Hiroyuki Yamada, Yoshihiko Ikeda, Toru Hashimoto

**Affiliations:** a Department of Cardiovascular Medicine, Narita-Tomisato Tokushukai Hospital, Chiba, Japan; b Department of Cardiology, Tokyo Medical University Hospital, Tokyo, Japan; c Department of Cardiovascular Surgery, Narita-Tomisato Tokushukai Hospital, Chiba, Japan; d Department of Pathology, National Cerebral and Cardiovascular Center, Suita, Japan

**Keywords:** Left ventricular pseudoaneurysm, Hypertrophic cardiomyopathy, Mid-ventricular obstruction, Left ventricular outpouching, Cardiac computed tomography angiography

## Abstract

**Background:**

Myocardial infarction-related left ventricular pseudoaneurysm (LVP), covered by the adjacent pericardial or scar tissue, is a fatal sequela of left ventricular rupture. Whereas hypertrophic cardiomyopathy (HCM) may cause left ventricular true aneurysm. Differentiating LVP from left ventricular true aneurysm is crucial because their natural histories and treatment strategies are distinct. However, the incidence and management of HCM-related LVP remain unknown.

**Case presentation:**

An 88-year-old man was admitted to our hospital with sudden-onset chest pain. Upon initial examination, vital signs were stable, and a grade 4/6 systolic murmur was noted. An electrocardiogram revealed atrial fibrillation and poor R-wave progression without ST-T changes or negative T-waves. An echocardiography showed mild left ventricular hypertrophy, mid-ventricular obstruction with a significant intraventricular pressure gradient, left ventricular outflow tract obstruction, and a small left ventricular apical outpouching. Cardiac computed tomography angiography (CCTA) assisted in the diagnosis of LVP, and an accompanying pericardial effusion suggested impending cardiac rupture. Because the patient initially refused our proposed urgent surgery, medication was initiated with continuous hemodynamic monitoring in the intensive care unit; however, the patient's condition did not improve. During a semi-urgent surgical repair of the aneurysmal wall, LVP was observed and confirmed by pathology. Myocardial tissue adjacent to the pseudoaneurysm was consistent with that of HCM. Subsequently, a final diagnosis of HCM-related LVP was made. The postoperative course was notable for transient profound hypotension. Thereafter, the patient died of non-occlusive mesenteric ischemia on day 6.

**Conclusions:**

To our knowledge, this is the first reported case of HCM-related LVP mimicking impending cardiac rupture. Our case highlights the importance of considering HCM-related LVP in patients with left ventricular outpouching and CCTA in the LVP diagnosis. In further research, data on the appropriate management of HCM-related LVP should be accumulated.

## Highlights

• Left ventricular pseudoaneurysm (LVP) can be fatal if left untreated.
• Hypertrophic cardiomyopathy (HCM) also may cause LVP.
• Cardiac computed tomography angiography is useful for the accurate diagnosis of LVP.
• The proper management for HCM-related LVP needs to be elucidated.

## 1 Introduction

Considering that left ventricular (LV) true aneurysms contain endocardium, epicardium, and fibrous tissue, left ventricular pseudoaneurysms (LVPs) lack endocardium and myocardium and are a fatal sequela resulting from cardiac rupture in the adherent pericardium or scar tissue. Because they are prone to rupture, they require early diagnosis and treatment [1]. LV true aneurysms occur in 8–15% of patients with myocardial infarction (MI) [2]. However, LVP is rare, occurring in 0.2–0.3% of patients following MI [3], although the exact incidence is unknown because of its lethal nature. The condition most frequently associated with LVP is transmural MI (55%), followed by cardiac surgery (33%), chest trauma (7%), and infection (5%) [1]. Other causes of LVP include catheter ablation procedures, Takayasu's arteritis, collagen disease, congenital heart disease, and idiopathic causes [[4], [5], [6], [7], [8]]. Early and accurate diagnosis remains challenging owing to the nonspecific signs and symptoms including chest pain, dyspnea, palpitations, and syncope. Consequently, it necessitates non-invasive advanced imaging methods for diagnosing LVP. Herein, we present the first reported case, to our knowledge, of hypertrophic cardiomyopathy (HCM)-related LVP.

## 2 Case presentation

### 2.1 Initial presentation

An 88-year-old man presented to the emergency department with sudden-onset chest pain. He had a history of hypertension and atrial fibrillation without prior syncope or stroke. He had no family history of HCM or sudden cardiac death and no history of cigarette smoking, alcohol abuse, or illicit drug use. He denied any history of chest trauma, recent MI, congenital heart disease, or catheter ablation procedures. In addition, he had no preceding flu-like symptoms. Pre-admission medication included azelnidipine (8 mg/day), olmesartan (10 mg/day), and cilostazol (50 mg/day). On admission, the patient's vital signs were as follows: blood pressure, 148/109 mmHg; heart rate, 77 beats/min; and body temperature, 36.6 °C. Physical examination revealed normal breath sounds and a grade 4/6 systolic murmur at the cardiac apex. No jugular venous distension, gallops, or leg edema was observed. Our initial concerns were acute MI and its mechanical complications, such as ventricular septal rupture and acute mitral regurgitation caused by papillary muscle rupture.

### 2.2 Diagnostic evaluation

**Fig. 1:**
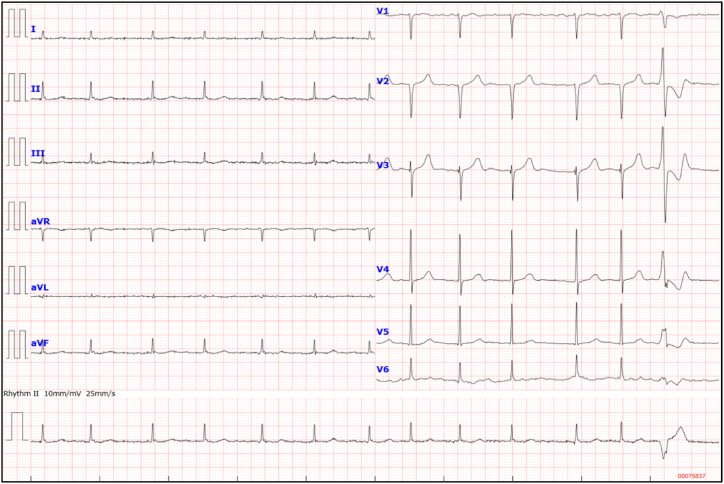
**Electrocardiogram on admission.** Electrocardiogram shows atrial fibrillation and poor R-wave progression with no remarkable ST-T changes or negative T-waves. Note the premature ventricular beat of a right bundle branch block with superior axis morphology.

**Fig. 2:**
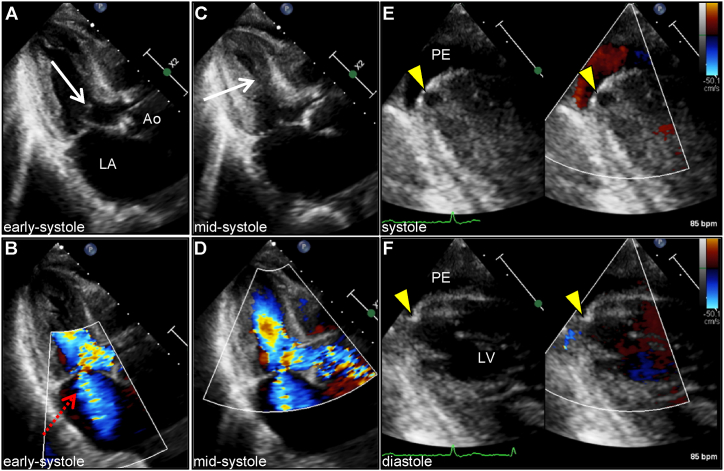
**Transthoracic echocardiography.** A transthoracic echocardiography reveals mild left ventricular hypertrophy (11 mm) and reduced chamber size (38 mm) with normal contraction (ejection fraction of 64%). Parasternal long-axis view reveals a sigmoid-shaped interventricular septum and an elongated anterior mitral leaflet with systolic anterior movement (arrow), resulting in septal contact leading to dynamic left ventricular outflow tract obstruction at early systole (A), concomitant with significant secondary mitral regurgitation with a posterior jet (dashed arrow) on color flow Doppler (B). It is notable that mid-cavity obstruction was observed (arrow) at mid-systole (C). Color Doppler echocardiography shows a peak flow velocity of 3.7 m/s at mid-systole, corresponding to a pressure gradient of 54 mmHg between the apex and basal of the LV (D). The modified apical four-chamber view shows an outpouching of the left ventricular apex that is remarkably visible during systole (arrowhead) and a moderate amount of pericardial effusion (E, F). The apparent flow is not recognized in apical outpouching by color Doppler. Ao, aorta; LA, left atrium; LV, left ventricle; PE, pericardial effusion. (For interpretation of the references to color in this figure legend, the reader is referred to the Web version of this article.)

Chest radiography was unremarkable. Electrocardiogram (ECG) revealed atrial fibrillation and poor R-wave progression but no remarkable ST-T changes or negative T-waves (Fig. 1). Notably, premature ventricular beat of a right bundle branch block with superior axis morphology was observed, suggesting a LV apex origin. His initial laboratory results showed mild anemia and elevated levels of C-reactive protein and brain natriuretic peptide (Table 1). The cardiac biomarker levels were within normal ranges. Echocardiography revealed a mild LV wall thickness and reduced chamber size with normal LV contraction. Sigmoid-shaped interventricular septum and mid-ventricular obstruction (MVO) coexistent with LV outflow tract obstruction were also recognized (Fig. 2A and C; and Video 1). Color Doppler echocardiography revealed a significant intraventricular pressure gradient (IVPG) between the apical and basal chambers of the left ventricle, and secondary mitral regurgitation (Fig. 2B and D; and Video 2). No remarkable intracardiac shunts were observed. Notably, a well-circumscribed small LV apical outpouching with a moderate amount of pericardial effusion was observed (Fig. 2E and F; and Video 3).

**Table 1:** Blood analyses.

Variables	On admission	Before surgery	Post-surgery	Day 6	Reference range
**Blood cell count**					
White blood cells (/μL)	56	76	108	20	33–86
Neutrophils (%)	66	77.4	87.2	89	38.5–80.5
Lymphocytes (%)	23	13.1	7.1	7.5	16.5–49.5
Red blood cells (/μL)	348	329	332	357	435–555
Hemoglobin (g/dL)	11.6	10.7	10.7	11.4	13.7–16.8
Hematocrit (%)	34.5	32.1	29.3	32.7	40.7–50.1
Platelet (10^4^/μL)	20.7	21.6	16.6	9.4	15.4–34.8
**Blood chemistry**					
Total protein (g/L)	6.5	6	5.2	5.2	6.6–8.1
Albumin (g/L)	3.6	3.4	3.2	3.7	4.1–5.1
Blood urea nitrogen (mg/dL)	23.8	34.3	24.7	53	8–20
Creatinine (mg/dL)	0.82	0.78	0.87	1.8	0.65–1.07
eGFR (mL/min/1.73 m^2^)	66.7	70.4	62.5	28.2	>60
Uric acid (mg/dL)	4.3	4.1	3.2	4.2	3.7–7.8
Total cholesterol (mg/dL)	173				160–220
Triglycerides (mg/dL)	45				50–150
High-density lipoprotein cholesterol (mg/dL)	79				>40
Low-density lipoprotein cholesterol (mg/dL)	85				70–140
Aspartate aminotransferase (U/L)	25	21	36	60	13–30
Alanine aminotransferase (U/L)	17	14	17	8	10–42
Lactate dehydrogenase (U/L)	220	193	271	258	124–222
Alkaline phosphatase (U/L)	243	230	185	132	106–322
γ-Glutamyl transferase (U/L)	25	22	27	26	13–64
Amylase (U/L)	83	56	64	73	44–132
Total bilirubin (mg/dL)	0.9	0.9	1.6	5.2	0.4–1.5
Creatine kinase (U/L)	73	70	284	290	59–248
Creatine kinase MB isoenzyme (U/L)	11	7	39	16	<25
Na (mmol/L)	141	136	133	144	138–145
K (mmol/L)	4.2	4.4	5.4	3.7	3.6–4.8
Cl (mmol/L)	107	106	103	108	101–108
C-reactive protein (mg/dL)	0.55	0.85	1.11	16.16	<0.30
Glucose (mg/dL)	85	110	197	45	73–109
Cardiac troponin I (pg/mL)	21		2377.5		<26.2
B-type natriuretic peptide (pg/mL)	125.8				<18.4
Hemoglobin A1c (%)	5.4				4.9–6.0
**Coagulation**					
Prothrombin time (sec)	11.8	12.3	13.7	23	<13.3
International normalized ratio	1.04	1.09	1.21	2.05	0.85–1.15
Activated partial thromboplastin time (sec)	30.4	32.1	36.8	152	<33.2
D-dimer (μg/mL)	1.1		1		<1.0

Abbreviation: eGFR, estimated glomerular filtration rate.

### 2.3 Differential diagnoses

**Fig. 3:**
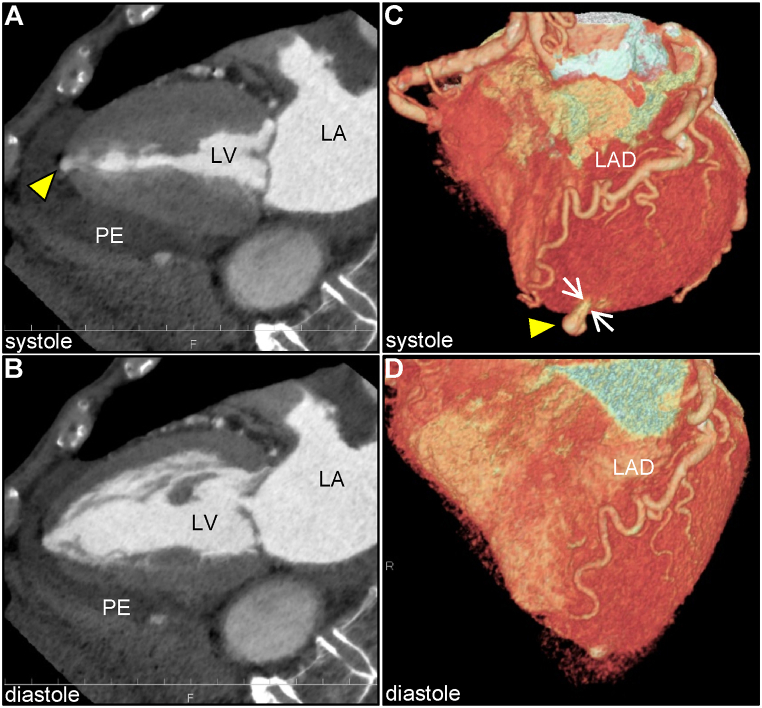
**Cardiac computed tomography angiography image of left ventricular apical outpouching.** Two-chamber view during systole (A) and diastole (B). Volume rendering image during systole (C) and diastole (D). It is notable that the apical outpouching with a narrow neck (arrows) of the LV caused significant protrusion during systole (arrowhead), and a moderate amount of pericardial effusion surrounding the LV. LA, left atrium; LAD, left ascending artery; LV, left ventricle; PE, pericardial effusion.

Potential diagnoses of LV apical outpouching included LV true aneurysm, LVP, diverticulum, cardiac hydatid cyst, and sub-epicardial cyst. Cardiac computed tomography angiography (CCTA) further characterized the morphology of the LV apical outpouching (Fig. 3A–D). CCTA revealed normal coronary arteries, but the presence of contrast-filled LV outpouching at the apex that protruded significantly during systole, with a maximum diameter of 5.4 mm, narrow orifice of 2.5 mm, and orifice-to-maximum diameter ratio of 46%, strongly suggested LVP. Notably, a moderate amount of pericardial effusion was observed. These findings along with the patient's symptoms were suggestive of LVP with impending cardiac rupture.

### 2.4 Management

The patient initially refused our proposed urgent surgery because of his advanced age. The patient was then transferred to the intensive care unit (ICU) for close monitoring. Lacking irrefutable evidence for HCM, we believed that chronic pressure overload within the MVO caused by the sigmoid septum and reduced LV chamber size resulted in an LVP at the LV apex. Preload reduction and tachycardia were risk factors for LV cavity narrowing, further increasing the IVPG in the MVO and promoting LVP expansion. After admission to the ICU, intravenous fluid replacement with 0.9% normal saline and continuous intravenous landiolol at a dose of 2 μg/kg/min were initiated to alleviate the load on the LVP by ameliorating the IVPG. Follow-up echocardiography revealed improvement of IVPG in MVO to 25 mmHg followed by reduced cardiac murmur on auscultation. The multidisciplinary team concluded that given the nature of LVP, which predisposes to cardiac rupture, and the superior long-term outcome of surgical intervention compared to that of conservative management [9] even in older patients, surgical repair of the LVP was required. The patient finally underwent a semi-urgent surgical repair of the apical aneurysm on day 3 after admission.

### 2.5 Surgical procedure

**Fig. 4:**
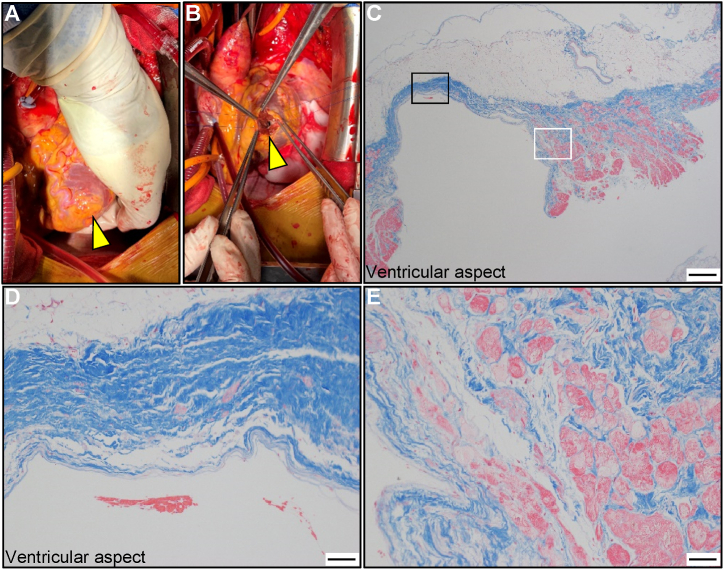
**Intraoperative and pathological findings.** Intraoperative view of the left ventricular pseudoaneurysm (arrowhead) (A). The aneurysm is opened (arrowhead) (B). Photomicrograph with Masson's trichrome staining (C–E): low-power view (C), bar: 500 μm; high-power view (D, magnified view of the black box in Fig. 3C), bar: 50 μm; and high-power view (E, magnified view of the white box in Fig. 3C), bar: 50 μm.

A median sternotomy with moderate hypothermic cardiopulmonary bypass using aorto-bicaval cannulation with aortic cross-clamping was performed. Eventually, the aneurysm was not adhered to the pericardium and was accompanied by serous pericardial fluid. The aneurysmal wall, approximately 7 mm in diameter, was vulnerable to thinning; however, there were no obvious signs of cardiac rupture (Fig. 4A and B). The apical aneurysm was repaired using vertical mattress sutures and reinforced with Teflon felt.

Pathologic examination of the excised specimen revealed transmural myocardial tissue depletion replaced with a thin fibrous membrane consistent with a pseudoaneurysm, and the adjacent LV myocardial tissue consisted of severe hypertrophy and disarrangement of myocytes with severe perivascular fibrosis (Fig. 4C–E), suggestive of HCM. Therefore, a final diagnosis of HCM-related LVP was made.

### 2.6 Outcome

**Fig. 5:**
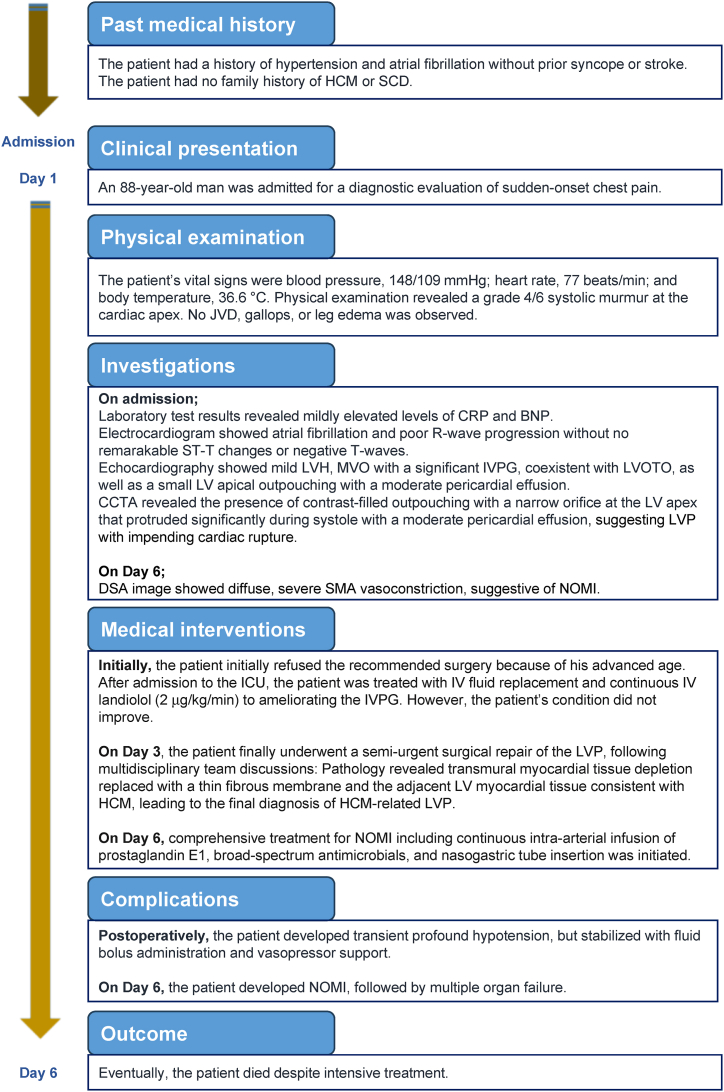
**Timeline of the patient's clinical course.** Abbreviations: BNP, brain natriuretic peptide; CCTA, cardiac computed tomography angiography; CRP, C-reactive protein; DSA, digital subtraction angiography; HCM, hypertrophic cardiomyopathy; ICU, intensive care unit; IV, intravenous; IVPG, intraventricular pressure gradient; JVD, jugular venous distension; LV, left ventricular; LVH, left ventricular hypertrophy; LVOTO, left ventricular outflow tract obstruction; LVP, left ventricular pseudoaneurysm; MVO, mid-ventricular obstruction; NOMI, non-occlusive mesenteric ischemia; SCD, sudden cardiac death; SMA, superior mesenteric artery.

Immediately following surgery, profound hypotension and tachycardia occurred because of preload reduction, which quickly resolved with intravenous fluid bolus administration of normal saline, and infusions of dopamine (10 μg/kg/min) and noradrenaline (0.5 μg/kg/min). A follow-up echocardiography revealed hyperkinetic LV contraction with a narrowed LV cavity, resulting in a significant MVO, presumably provoked by tachycardia and the need for continuous intravenous landiolol (5 μg/kg/min). Close hemodynamic monitoring of arterial pressure continued in the ICU. The patient was hemodynamically stable thereafter. On postoperative day 3 (hospitalization day 6), the patient experienced nausea and vomiting. He developed shock with a systolic blood pressure of 70 mmHg, requiring intravenous administration of isotonic saline solution and noradrenaline (0.5 μg/kg/min). In addition, the patient developed severe hypoxemia with a SpO2 of 88%, requiring tracheal intubation for respiratory insufficiency. Arterial blood gas analysis revealed severe acidemia (pH 7.248, pO_2_ 53 mmHg, pCO_2_ 55 mmHg, BE -3.7) with an increased serum lactate level of 5.5 mmol/L (reference: 0.5–1.5 mmol/L). Abdominal distention was observed. Enhanced abdominal computed tomography suggested acute small bowel ischemia characterized by a severe functional ileus, raising suspicion of non-occlusive mesenteric ischemia (NOMI). Although we strongly recommended emergent abdominal surgery, the family refused our proposal. Selective angiography of the superior mesenteric artery was performed. Digital subtraction angiographic images revealed severe diffuse narrowing of the superior mesenteric artery and its branch vessels, with a reduced number of mesenteric vessels, suggesting vasospasm (data not shown). A constellation of clinical features, exclusion of embolic mesenteric artery occlusion, and radiographic findings fulfilled the diagnostic criteria for NOMI [10]. To reverse mesenteric vasoconstriction, prostaglandin E1 was administered as an initial bolus of 10 μg and a subsequent continuous intra-arterial infusion of 80 μg/day. In addition, supportive care, including intravenous broad-spectrum antimicrobials (ceftriaxone, 2 g twice daily) and insertion of a nasogastric tube to achieve gastric decompression, was provided. The patient's condition worsened thereafter and was complicated by multiple organ failure (Table 1). Eventually, the patient died despite intensive treatment. Fig. 5 shows a timeline of the patient's clinical course.

## 3 Discussion

Here, to the best of our knowledge, we report the first case of HCM-related LVP. Our case may provide the following clinical key points.

### 3.1 Diagnostic considerations

Compared with LV true aneurysms, LVPs have the following distinct morphological and histological characteristics (Table 2) [1,11,12,and^13^]: they occur predominantly in the posterior and lateral LV walls; the aneurysm orifice is narrower than the maximum diameter of the aneurysm; sudden destruction of myocardial tissue at the transition between the LV wall and the aneurysm; the aneurysmal wall is composed of tissue without myocardial tissue. It is difficult to visualize the LV apical area in some cases because of poor spatial resolution by echocardiography [14]. Actually, the size of the LVP was too small for echocardiography to clearly visualize the pseudoaneurysm at the LV apex in our case. Among the differential diseases causing LV outpouchings, CCTA was useful in differentiating each of them based on the size of the orifice across which the LV outpouching communicates with the left ventricle and the movement of the bulge during a cardiac cycle [15]. LV true aneurysm has a wide neck and expands paradoxically during systole, whereas LVP has a narrow orifice with paradoxical expansion during systole, which was helpful for establishing LVP diagnosis in this case.

**Table 2:** Morphological and histological differences between left ventricular true aneurysm and pseudoaneurysm.

	True aneurysm	Pseudoaneurysm	
**Morphological features**			
Location	Anterior or apical walls	Posterior or lateral walls	
Opening to the aneurysm	Broad neck	Narrow neck	
Orifice/Aneurysm diameter ratio	>0.5	<0.5	
Transition site of the aneurysm	No interruption	Abrupt interruption	
Aneurysmal wall motion	Akinetic or dyskinetic during systole	Dyskinetic during systole	
**Histological features**			
Wall component	Thinned out myocardial scar	Adherent pericardium or scar tissue	
Endocardium and myocardium	Remaining	None	
Endocardial continuity	Maintained	Disrupted	

Notably, our patient with HCM presented without detectable ECG findings characterizing HCM. The ECG is recommended as a screening tool for HCM because it yields a high diagnostic value for identifying HCM with a LV wall thickness >13 mm as echocardiography (sensitivity and specificity: 61% and 97% for ECG and 62% and 100% for echocardiography, respectively) [16]. However, a large study of 2,485 patients with HCM reported that normal ECG findings for HCM occurred in approximately 6% of patients with documented HCM [17]. Considering that the prevalence of HCM was 0.2%, the incidence of normal ECG findings in patients with HCM corresponded to 0.01% in general screening, suggestive of its extreme rarity. In our case, mild LV wall thickness, no family history of HCM, and the absence of detectable ECG findings typical of HCM might have interfered in establishing a definite diagnosis of HCM-related LVP. Therefore, our case highlights the importance of being aware of the specific subgroup of patients with HCM showing normal ECG findings.

### 3.2 Management strategies

Our case raises the clinical issue of proper management for HCM-related LVP.

Surgical repair is often considered the first-line treatment for MI-related LVP, despite a high surgical mortality rate of 7%–23% [18], based on the histological fragility of LVP and the previously reported high risk of cardiac rupture (30%–45%) in patients with non-surgically treated MI-related LVP [1,19]. Percutaneous embolization is an alternative therapeutic option for high-risk surgical patients [20]. In certain circumstances, such as asymptomatic cases, small aneurysmal diameters, and chronic phase, conservative management can be another feasible treatment option to maintain a low risk of cardiac rupture [21]. Currently, no evidence-based treatment strategies exist for HCM-related LVP. The most sensitive symptoms and signs preceding impending cardiac rupture may include two or more symptoms, including pericardial pain, repetitive nausea, restlessness, agitation, abrupt and transient hypotension, unexpected T-wave alterations on ECG, or echocardiographic features of pericardial effusion >5 mm [22,23]. Based on the imaging findings characteristic of LVP, coexistent pericardial effusion, and sudden-onset chest pain, we clinically judged that the risk of short-term cardiac rupture was high in the present case. Considering the patient's old age, we opted for a minimally invasive surgical repair of LVP instead of extended radical surgery for possible MVO-HCM. However, the postoperative course was eventful for NOMI with unfavorable outcomes. Although multiple factors might have triggered NOMI in our case, the residual IVPG might have been an indirect factor for inducing NOMI. Medical treatment could have been an alternative option in our case. Hence, collecting evidence on the best approach for HCM-related LVP is needed.

### 3.3 Clinical implications

Our patient presented with a histologically-proven HCM-related LVP characterized by the complete depletion of both myocytes and endocardium, which was replaced with fibrosis. However, LVP was free from the pericardium. Furthermore, the negative findings of the pericardial tissue and thrombus in the LVP were distinct from the findings observed in typical MI-related LVP. LV apical aneurysm can form in the clinical course of HCM, with an incidence of 2%–4.8% of patients with HCM [14,24]. Generally, HCM-related LV apical aneurysms are considered true aneurysms, in which cardiomyocytes are gradually heterogeneously depleted and replaced by collagen fibers, providing a fertile nidus for the development of severe ventricular arrhythmias, mural thrombus, or systemic thromboembolism [25,26]. Several factors involved in chronic myocardial ischemia may affect true apical aneurysm formation in patients with HCM: oxygen supply demand mismatch due to severe myocardial hypertrophy and thickened intramural arteries, transient coronary artery occlusion due to myocardial bridging, and impaired coronary circulation due to an increased mid-ventricular gradient [27]. However, the association between HCM and LVP has not yet been reported. LVP observed in our case and HCM-related true aneurysms showed apparently distinct histological characteristics and morphological and functional features on imaging. In addition, the possibility of a ruptured LV sub-epicardial cyst could not be ruled out until a histologic diagnosis was made [28]. Whether the LVP observed in our case was a continuum of common HCM-related true aneurysms remains unclear. Further research on its underlying mechanism and natural history is warranted.

### 3.4 Limitations

The present case report had three limitations.

First, cardiac magnetic resonance is a reliable diagnostic alternative to facilitate morphological and functional evaluation of LV outpouchings as well as their tissue characterization. Generally, the late gadolinium enhancement image shows no pericardial enhancement in the LV true aneurysm. Whereas, pericardial enhancement reflecting the inflammatory response and pericardial angiogenesis is often observed in patients with LVP [29]. In this case, CCTA was preferable to cardiac magnetic resonance imaging, requiring a longer acquisition time, because the patient's condition was considered potentially hemodynamically unstable.

Second, although various sarcomere gene mutations encoding components of the contractile apparatus have been reported in cases of HCM [30], genetic testing could not be performed in this case.

Finally, surgical repair was performed to allow safe and reliable resection of the LVP in this case. Percutaneous repair was not the first choice because of the technical difficulty of approaching the LV apex due to the patient's highly deformed left ventricle and the uncertain long-term outcome. However, given the severe postoperative hypotension and subsequent fatal complications, a less invasive percutaneous embolization might have been more appropriate in this case.

## 4 Conclusions

To the best of our knowledge, we describe the first case of a patient with HCM-related LVP that required early surgical intervention, but eventually died from a non-cardiogenic cause. HCM can cause not only a true apical aneurysm but also LVP. Therefore, CCTA should be performed in patients with HCM with LV outpouching. LVP may have been left undetected as a possible cause of sudden cardiac death in patients with HCM. Although the exact mechanism remains unclear, the natural history, triggers, and management of HCM-related LVP need to be examined in the future.

## Ethical statement

*Consent:* The authors confirm that written consent for submission and publication of this case report including images and the associated videos has been obtained from the patient.

## Data availability statement

Data included in article/supplementary material/referenced in article.

## Funding statement

This research did not receive any specific grant from funding agencies in the public, commercial, or not-for-profit sectors.

## CRediT authorship contribution statement

**Katsuya Hashimoto:** Investigation, Data curation. **Hiroyuki Yamamoto:** Writing – review & editing, Writing – original draft, Visualization, Investigation, Data curation, Conceptualization. **Atsushi Harada:** Data curation. **Hiroyuki Yamada:** Writing – original draft, Supervision, Data curation. **Yoshihiko Ikeda:** Writing – review & editing, Writing – original draft, Visualization, Validation, Supervision, Methodology, Investigation, Conceptualization. **Toru Hashimoto:** Supervision, Data curation.

## Declaration of competing interest

The authors declare that they have no known competing financial interests or personal relationships that could have appeared to influence the work reported in this paper.

## Appendix A Supplementary data

Video 1 Transthoracic echocardiography in apical three-chamber view. Video 1

Video 2 Color Doppler transthoracic echocardiography in apical three-chamber view. Video 2

Video 3 Transthoracic echocardiography in modified apical two-chamber view with and without color Doppler mode. Video 3
